# Effects of propofol on neuroblastoma cells via the HOTAIRM1/miR-519a-3p axis

**DOI:** 10.1515/tnsci-2022-0212

**Published:** 2022-03-10

**Authors:** Guan Wang, Yao Yu, Yang Wang

**Affiliations:** Department of Anesthesiology, The Second Hospital of Dalian Medical University, No. 467 Zhongshan Road, Shahekou District, Dalian, Liaoning, China

**Keywords:** MPP^+^, propofol, HOTAIRM1, miR-519a-3p, neuroblastoma cells

## Abstract

**Background:**

Propofol, an intravenous sedative-hypnotic agent, is demonstrated to have antioxidant properties. The purpose of this study is to investigate the functional roles of propofol in neuroblastoma cells.

**Methods:**

The proliferation and apoptosis were assessed by 3-(4,5-dimethylthiazol-2-yl)-2, 5-diphenyl-2*H*-tetrazol-3-ium bromide (MTT), EdU, and flow cytometry assays, respectively. The protein expression level was quantified by western blot assay. Inflammation and oxidative stress were determined by measuring the release of inflammatory factors, along with intracellular reactive oxygen species (ROS), lactate dehydrogenase (LDH), malondialdehyde (MDA), and superoxide dismutase (SOD) levels. The real-time quantitative polymerase chain reaction (RT-qPCR) was conducted to assess the expression levels of HOXA transcript antisense RNA, myeloid-specific 1 (HOTAIRM1), and miR-519a-3p in cells. The interaction relationship between HOTAIRM1 and miR-519a-3p was confirmed by dual-luciferase reporter, RNA immunoprecipitation (RIP), and RNA pull-down assays.

**Results:**

Treatment with MPP^+^ has been observed to induce apoptosis, oxidative stress, and inflammation in neuroblastoma cells, which were abolished by propofol or silencing of HOTAIRM1. Importantly, the increase of HOTAIRM1 and the decrease of miR-519a-3p caused by MPP^+^ were reversed by propofol in neuroblastoma cells. In addition, miR-519a-3p was a target of HOTAIRM1, and inhibition of miR-519a-3p abolished HOTAIRM1 silencing-induced effects on neuroblastoma cells. Moreover, functional experiments revealed that propofol might weaken MPP^+^-induced apoptosis, oxidative stress, and inflammation by regulating the HOTAIRM1/miR-519a-3p axis.

**Conclusion:**

Propofol inhibited oxidative stress and inflammation in MPP^+^-induced neuroblastoma cells by targeting the HOTAIRM1/miR-519a-3p axis, implying the potential protective function of propofol against oxidative damage.

## 1 Introduction

Parkinson’s disease (PD) is an age-related and long-term worsening disease in the central nervous system, resulting in resting tremor, increased muscle tone, rigidity, and sluggishness [1]. Although intensive research studies have shown that different environmental factors can influence the development of PD, the exact etiology and pathogenesis of PD are complex, and still not well understood [2]. The dysfunction and the pathophysiologic loss of dopaminergic neurons in the midbrain substantial nigra were the main pathological changes in the PD process, triggering a decrease of the striatum [3,4]. Furthermore, 1-methyl-4-phe-nylpyridinium (MPP^+^) was used to establish the PD model *in vitro*, replicating most of the pathophysiological changes in PD, such as neurons injury, inflammation, and oxidative stress [5].

Propofol is one of the most commonly used anesthetics. It has been proposed that in addition to the anesthetic properties, the powerful immunomodulatory, antioxidant, analgesic and neuroprotective properties of propofol are particularly noteworthy [6]. Propofol can reinforce the antioxidative system to protect tissues, such as the brain, liver, kidneys, lungs, and heart, against oxidative stress [7,8]. Thus, we speculated that propofol might have roles in PD prevention.

Long non-coding RNAs (lncRNAs) were reported to act vital regulatory roles in various diseases through regulating gene expression [9]. The dysregulation of HOXA transcript antisense RNA, myeloid-specific 1 (HOTAIRM1) was found in a variety of human malignancies, including endometrial cancer [10], colorectal cancer [11], and glioma [12]. Besides, HOTAIRM1 was reported to be implicated in neurogenesis [13]. Fan et al. showed that HOTAIRM1 was highly expressed in PD patients, and had potential value for PD diagnosis; moreover, HOTAIRM1 expression was also increased in 6-hydroxydopamine (6-OHDA)-stimulated SH-SY5Y cells, *in vitro* system used in the PD study, and reduced cell viability, suggesting the involvement of HOTAIRM1 in the pathogenesis of PD [14]. Therefore, the regulatory roles of HOTAIRM1 in PD were investigated in this study. MicroRNAs (miRNAs) are defined as nontranslated transcripts generally with 18-23 nucleotides in length, and miRNAs play regulatory roles in the modulation of gene expression through interaction with target mRNAs [15]. Leggio et al. reported that miRNAs were associated with the expression of PD-related genes, suggesting the diagnostic and therapeutic values of miRNAs in PD [16]. As an example, microRNA 7 could slow the development of PD by keeping neurons activity to some extent [17]. miR-519a-3p is a functional miRNA. The upregulation of miR-519a-3p was involved in the poor survival in breast cancer patients, and overexpression of miR-519a-3p was contributed to the aggressive phenotype of breast cancer cells, which decreased cell apoptosis [18]. Furthermore, miR-519a-3p was identified to be differentially expressed in PD when compared with healthy controls [19]. Here, we speculated that miR-519a-3p dysregulation might be associated with the pathogenesis of PD.

Previous studies have shown that propofol can affect epigenetic pathways, such as those involving lncRNA and miRNA to exert its properties [20,21]. Herein, we used a cell model for PD via incubating neuroblastoma cells with 1-methyl-4-phe-nylpyridinium (MPP^+^) to investigate the functional roles of propofol in the PD process. Besides, we also elucidated whether there was a regulatory relationship among propofol, HOTAIRM1, and miR-519a-3p in MPP^+^-induced neuroblastoma cells.

## 2 Materials and methods

### 2.1 Cell lines and cell culture

Human neuroblastoma cell lines (SK-N-SH and SK-N-AS) were purchased from the American Type Culture Collection (Rockville, MD, USA). SK-N-SH and SK-N-AS cells were propagated in RPMI 1640 medium (Biochrom KG, Berlin, Germany) containing 10% fetal bovine serum (Thermo Fisher Scientific, Carlsbad, CA, USA) and penicillin/streptomycin (Biochrom KG) in an incubator with 5% CO_2_ at 37°C. In addition, MPP^+^ and propofol were both purchased from Sigma-Aldrich (Louis, Missouri, USA).

### 2.2 Cell proliferation assay

For 3-(4,5-dimethylthiazol-2-yl)-2, 5-diphenyl-2*H*-tetrazol-3-ium bromide (MTT) assay, SK-N-SH and SK-N-AS cells were planted into 96-well plates (4 × 10^3^ cells/well) with 5% CO_2_ at 37°C. After 48 h, a total of 20 µL of MTT solution (Sigma-Aldrich) was subsequently added into each well and incubated for additional 4 h. A total of 150 µL of dimethyl sulfoxide (DMSO, Sigma-Aldrich) was administered to cells to dissolve the formazan crystals in 96-well plates. The absorbance rate (wavelength: 490 nm) was assessed under a spectrophotometer (Molecular Devices, Sunnyvale, CA, USA). For EdU (5-ethynyl-2′-deoxyuridine) assay, 1 × 10^4^ cells were inoculated to 24-well plates and stained as per the instructions of the EdU cell proliferation kit (Ribobio, Guangzhou, China). The EdU-positive cells were counted in five random fields per well.

### 2.3 Cell apoptosis assay

For cell apoptosis assay, neuroblastoma cells were re-suspended with phosphate buffer saline buffer solution (1 × 10^6^/mL). About 1 mL of cell suspension in the tube was added with staining buffer containing 5 µL of fluorescein isothiocyanate (FITC) and 5 µL of propidium iodide (BestBio, Shanghai, China). After incubation in dark conditions for 30 min, cells were collected and subjected to apoptosis analysis under flow cytometry (Applied Biosystems, Foster City, CA, USA).

### 2.4 Western blot assay

Briefly, neuroblastoma cells were collected and lysed using adio-immunoprecipitation assay (RIPA) buffer (Cell Signaling Technology, Danvers, MA, USA), followed by quantifying the lysates using the bicinchoninic acid (BCA) protein assay (Sigma-Aldrich). Samples (40 µg of protein) were fractionated by sodium dodecyl sulfate polyacrylamide gel electrophoresis, followed by transferring into nitrocellulose membranes (Bio-Rad, Hercules, CA, USA). The membranes were incubated with primary antibodies at 4°C for 24 h. After being washed with Tris-buffered saline in Tween 20, membranes were reacted with secondary antibody with HRP-conjugated (ab1500771; 1:3,000 dilution; Abcam, Cambridge, MA, USA) for 2 h. Finally, the signals were determined by a chemiluminescence detection kit (Pierce Biotechnology, Rockford, IL, USA). The primary antibodies were as follows: B-cell lymphoma-2 (Bcl-2; ab59348; 1:2,000 dilution; Abcam), cleaved caspase-3/total-caspase 3 (C-caspase3/t-caspase 3; ab184787; 1:2,000 dilution; Abcam), BCL2-associated X (Bax; ab32503; 1:2,000 dilution; Abcam), and glyceraldehyde-3-phosphate dehydrogenase (GAPDH) (ab181602; 1:2,000 dilution; Abcam).

### 2.5 Enzyme-linked immunosorbent assay (ELISA)

The content of tumor necrosis factor-α (TNF-α) and interleukin-1β (IL-1β) in the supernatants of neuroblastoma cells was measured with an ELISA kit (Invitrogen, Carlsbad, CA, USA). Briefly, 50 µL of the cell supernatant was collected and then added into a 96-well plate treated with goat anti-mouse IgM. After incubating at 37°C for 60 min, the substrate was added to 96-well plates. After stopping the reaction, the absorbance was read at 492 nm with a spectrophotometer (Molecular Devices).

### 2.6 Oxidative stress assay

SK-N-SH and SK-N-AS cells were collected and lysed with 0.2% Triton X-100 (Sigma-Aldrich). After centrifugation, 50 µL of the supernatant was collected for the next experiments. Lactate dehydrogenase (LDH) Cytotoxicity Detection Kit (Thermo Fisher Scientific) was used to assess the intracellular LDH level. Similarly, the activity levels of superoxide dismutase (SOD) and malondialdehyde (MDA) were checked using the SOD Assay Kit or Cell MDA assay kit (Invitrogen). In addition, reactive oxygen species (ROS) generation was assessed with ROS Assay Kit (Invitrogen) referring to the recommended protocol.

### 2.7 Total RNA extraction and real-time quantitative polymerase chain reaction (RT-qPCR) assay

Total RNA was isolated from SK-N-SH and SK-N-AS cells with the Trizol reagent (Invitrogen) in the light of the manufacturer’s protocols. RNA was quantified with a NanoDrop ND-1000 spectrophotometer (Thermo Fisher Scientific). Total RNA was reverse-transcripted into complementary DNA using a PrimeScript™ RT reagent kit (CapitalBio, Beijing, China) or TaqMan microRNA reverse transcription kit (Applied Biosystems). The RT-qPCR assay was performed with FastStart Universal SYBR-Green Master Mix (Roche, Basel, Switzerland) or TaqMan MicroRNA Assays (Applied Biosystems) with the Roche LC480 system (Roche Applied Science, Mannheim, Germany) based on the 2^−ΔΔCt^ method. The expression level of HOTAIRM1 was standardized to GAPDH, and miR-519a-3p expression was normalized to endogenous small nuclear RNA U6.

The sequences of primers:

HOTAIRM1 (sense, 5′-CATTTAAATCCCCGGCGCTC-3′; antisense, 5′-TTCGCTCCAGCACTCCAAAT-3′);

miR-519a-3p (sense, 5′-GCCGAGAAAGTGCATCCTT-3′; antisense, 5′-CTCAACTGGTGTCGTGGA-3′);

GAPDH (sense, 5′-TCCCATCACCATCTTCCAGG-3′; antisense, 5′-GATGACCCTTTTGGCTCCC-3′);

U6 (sense, 5′-AACGCTTCACGAATTTGCGT-3′; antisense, 5′-CTCGCTTCGGCAGCACA-3′).

### 2.8 Transfection assay

miR-519a-3p mimic and its control (NC), and miR-519a-3p inhibitor (anti-miR-519a-3p) and its control (anti-NC) were purchased from Biosci Company (Wuhan, China). Small interfering RNA (siRNA) targeted HOTAIRM1 (si-HOTAIRM1), scrambled control (si-NC), HOTAIRM1-overexpression vector (HOTAIRM1), and negative control (Lnc-NC) were constructed by GeneCopoeia (Rockville, MD, USA). Neuroblastoma cells were seeded into 6-well plates (1 × 10^5^ cells/well) and incubated for 24 h; then the above oligonucleotides and vectors alone or in combination were transfected into neuroblastoma cells by Lipofectamine 2000 (Thermo Fisher Scientific).

### 2.9 Dual-luciferase reporter assay

The possible binding sites of HOTAIRM1 and miR-519a-3p were shown by LncBase Predicted v.2 (http://carolina.imis.athena-innovation.gr/diana_tools/web/index.php?r=lncbasev2%2Findex-predicted&miRNAs%5B%5D=hsa-miR-519a-3p&lncRNAs%5B%5D=&lncRNAs%5B%5D=ENSG00000233429&threshold=0.7&filters=0). HOTAIRM1 fragments containing either the predicted potential miR-519a-3p binding sites wild-type (HOTAIRM1-wt) or site sequences mutation (HOTAIRM1-mut) were cloned into the pGL3-basic vectors (Realgene, Nanjing, China). SK-N-SH and SK-N-AS cells (2 × 10^5^ cells/well in 24-well plates) were co-transfected with 20 pmol of miR-519a-3p mimic or NC and vector containing HOTAIRM1-wt or HOTAIRM1-mut. Cells were harvested 48 h later, and then the luciferase signal was checked under Dual-Luciferase Reporter Assay System (Thermo Fisher Scientific) and standardized to Renilla luciferase activity.

### 2.10 RNA immunoprecipitation (RIP) and RNA pull-down assay

The neuroblastoma cells were lysed with Imprint^®^ RNA immunoprecipitation kit (Sigma-Aldrich). After that, 200 µL of the lysate was incubated with magnetic beads embracing Ago2 or IgG antibodies at 4°C overnight. After centrifuging, immunoprecipitated RNA was incubated with proteinase K treatment, followed by RT-qPCR assay. For RNA pull-down assay, lysates from neuroblastoma cells were mixed with 100 pmol of miR-519a-3p harboring biotinylated RNA (bio-miR-519a-3p) or bio-NC (control). After incubation at room temperature for 1 h, biotin-miRNA-lncRNA complexes were pulled down and then extracted with the Trizol reagent (Invitrogen). The expression of HOTAIRM1 was determined with RT-qPCR assay.

### 2.11 Statistical analysis

Statistical analyses were conducted using SPSS 21.0 software (IBM, Somers, NY, USA). For multiple group comparisons, analysis of variance followed by Tukey *post hoc* testing was performed. The student’s *t*-test was used for the comparisons of two groups. Data are expressed as mean ± standard deviation. A *P*-value less than 0.05 was considered to indicate a significant difference. *P < 0.05 vs the corresponding control.

## 3 Results

### 3.1 Propofol could weaken apoptosis, inflammation, and oxidative damage in neuroblastoma cells caused by MPP^+^

**Figure 1:**
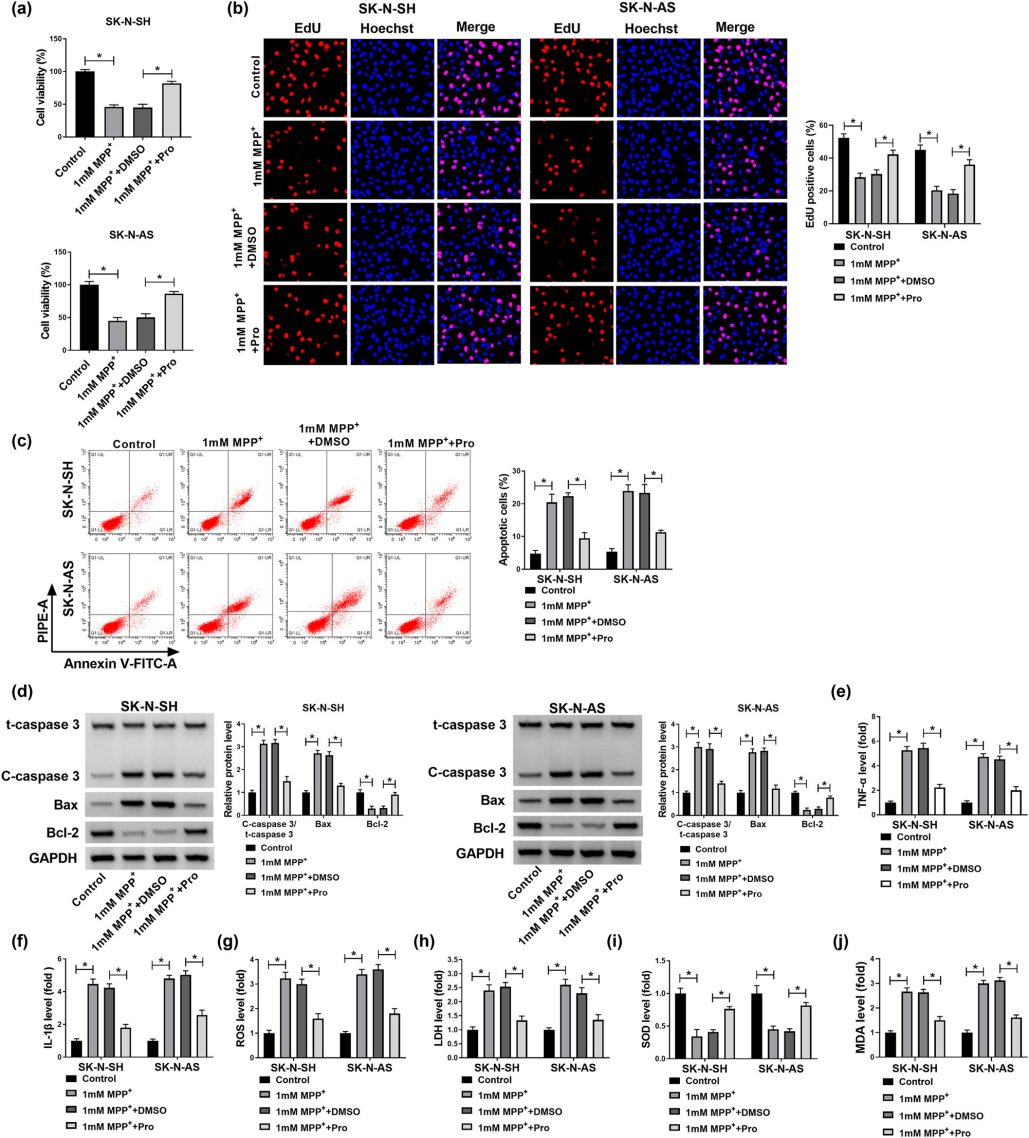
Effects of propofol on proliferation, apoptosis, inflammation, and oxidative stress of neuroblastoma cells treated with MPP^+^. (a–h) SK-N-SH and SK-N-AS cells were treated with 1 mM MPP^+^, 1 mM MPP^+^ + DMSO, 1 mM MPP^+^ + Pro, with untreated cells as the Control group. (a and b) The MTT and EdU assays were applied to detect cell proliferation in SK-N-SH and SK-N-AS cells. (c) Apoptotic cells were quantified by flow cytometry assay. (d) The levels of C-caspase3/t-caspase3, Bax, and Bcl-2 were shown by western blot assay. (e and f) The relative levels of TNF-α and IL-1β were analyzed by commercial kits in the supernatant. (g–j) The intracellular ROS generation, LDH activity, SOD content, and MDA level were measured by commercial kits. *P < 0.05.

MPP^+^ was used to induce the PD model *in vitro* by involving cell death. As shown in Figure S1a and b, the viability of SK-N-SH and SK-N-AS cells treated with increasing concentrations of propofol was investigated at 1 mM MPP^+^, and the results showed that cell viability was dose-dependently elevated by propofol in 1 mM MPP^+^-induced SK-N-SH and SK-N-AS cells; moreover, 50 µM propofol was selected for subsequent analysis due to about 50% of cell viability. Then, as presented in Figure 1a, 50 µM propofol could rescue cell viability arrest in SK-N-SH and SK-N-AS cells induced by treatment with 1 mM MPP^+^. Subsequent EdU analysis suggested that propofol treatment rescued MPP^+^-caused inhibition of DNA synthesis activity in SK-N-SH and SK-N-AS cells (Figure 1b). The analysis of flow cytometry assay revealed that propofol protected SK-N-SH and SK-N-AS cells from MPP^+^-induced apoptosis (Figure 1c). Likewise, the ratio of C-caspase3/t-caspase3 and Bax expression were increased, while Bcl-2 was decreased in SK-N-SH and SK-N-AS cells after treatment with 1 mM MPP^+^, which were overturned by treatment with propofol (Figure 1d). Propofol could inhibit inflammation in MPP^+^-treated cells by decreasing TNF-α and IL-1β (Figure 1e and f). MPP^+^ induced oxidative damage by increasing ROS, LDH, and MDA levels while decreasing SOD activity, which was abolished by propofol in SK-N-SH and SK-N-AS cells (Figure 1g–j). These results revealed that MPP^+^-caused apoptosis, inflammation, and oxidative damage were overturned by propofol in SK-N-SH and SK-N-AS cells.

### 3.2 HOTAIRM1 was upregulated while miR-519a-3p was downregulated in neuroblastoma cells exposed to MPP^+^

**Figure 2:**
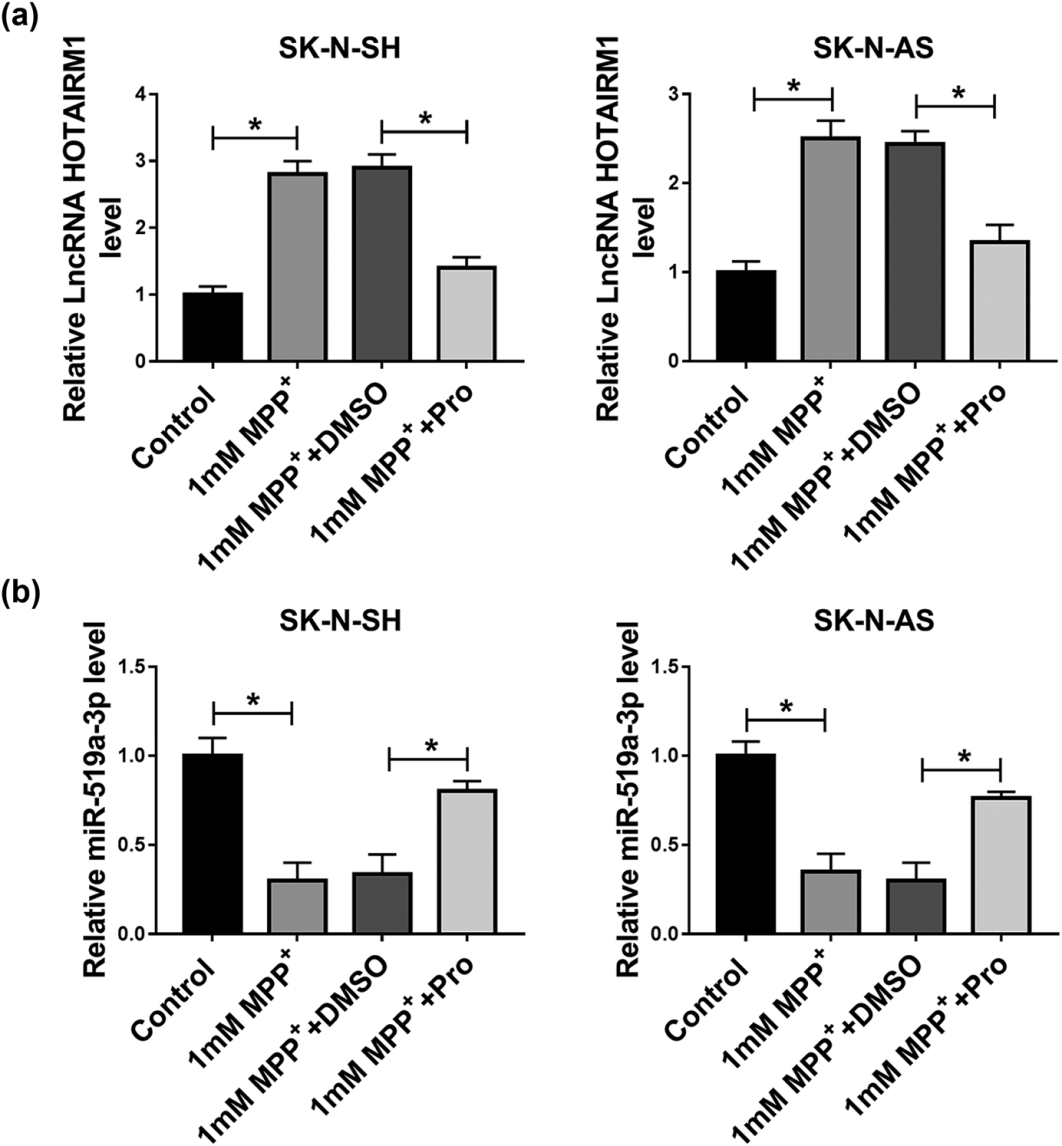
The expression level of HOTAIRM1 and miR-519a-3p in neuroblastoma cells. (a and b) The RT-qPCR assay was employed to measure the expression level of HOTAIRM1 and miR-519a-3p in SK-N-SH and SK-N-AS cells treated with 1 mM MPP^+^, 1 mM MPP^+^ + DMSO, 1 mM MPP^+^ + Pro, with untreated cells as the Control group. *P < 0.05.

To determine whether HOTAIRM1 and miR-519a-3p were involved in MPP^+^-caused inflammation and oxidative damage, RT-qPCR assay was performed in SK-N-SH and SK-N-AS cells. The upregulation of HOTAIRM1 induced by MPP^+^ was abolished by propofol in SK-N-SH and SK-N-AS cells (Figure 2a). In addition, the treatment with propofol could counteract MPP^+^ induced- downregulation of miR-519a-3p by RT-qPCR assay (Figure 2b). Therefore, HOTAIRM1 and miR-519a-3p might play key roles in the protective effects of propofol on SK-N-SH and SK-N-AS cells.

### 3.3 Knockdown of HOTAIRM1 abolished MPP^+^-induced effects on neuroblastoma cells

**Figure 3:**
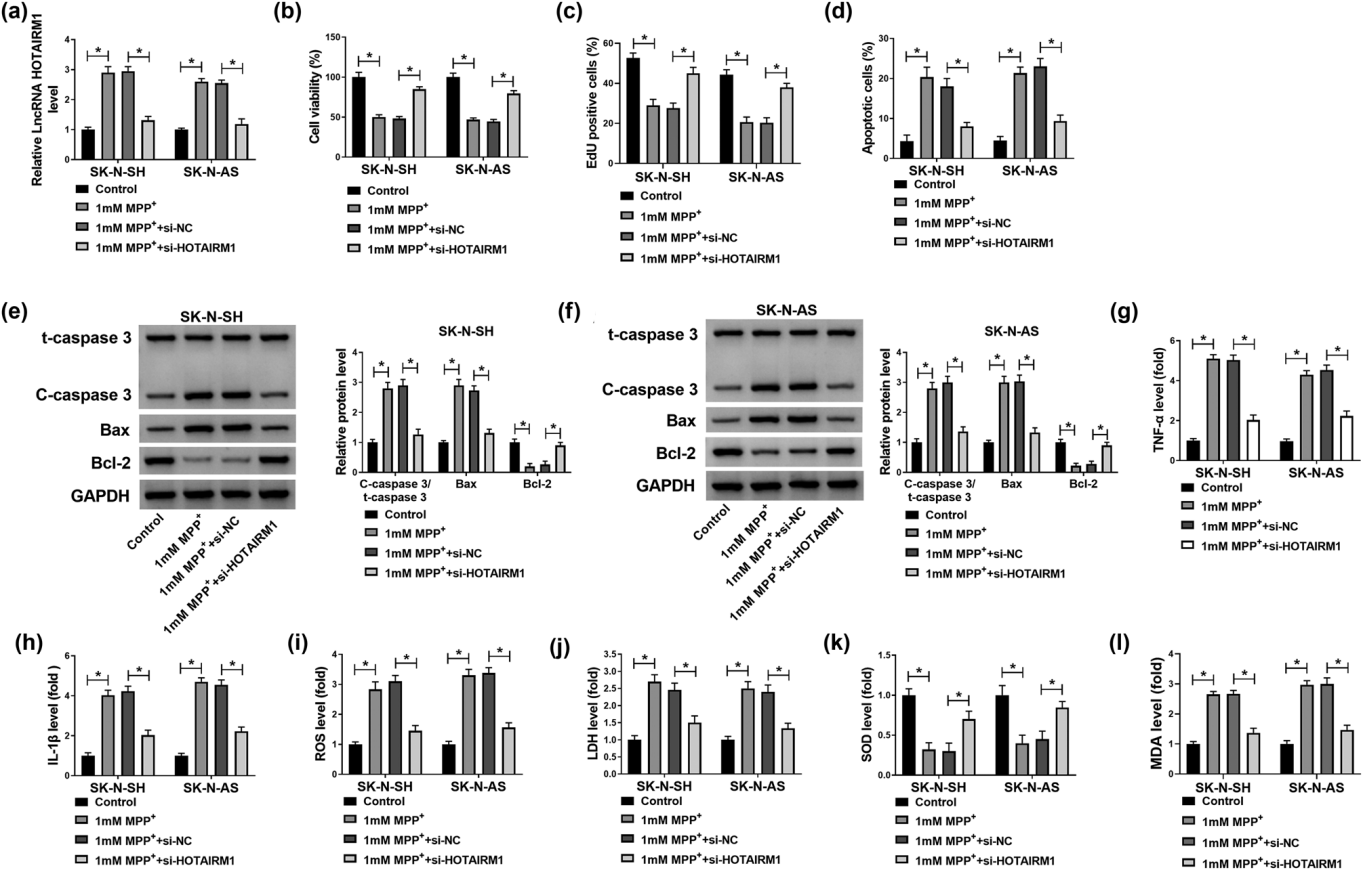
Effects of HOTAIRM1 silencing on proliferation, apoptosis, inflammation, and oxidative stress in neuroblastoma cells treated with MPP^+^. (a–j) SK-N-SH and SK-N-AS cells were treated with 1 mM MPP^+^, 1 mM MPP^+^ + si-NC, and 1 mM MPP^+^ + si-HOTAIRM1, with untreated cells as the Control group. (a) The expression level of HOTAIRM1 was determined by RT-qPCR assay. (b and c) The proliferation of SK-N-SH and SK-N-AS cells was measured by MTT and EdU assays. (d) Flow cytometry assay was employed to measure the apoptosis of SK-N-SH and SK-N-AS cells. (e and f) Western blot assay was performed to assess the levels of C-caspase3/t-caspase3, Bax, and Bcl-2. (g–l) The levels of TNF-α and IL-1β in the supernatant and intracellular ROS generation, LDH activity, SOD content, and MDA level were analyzed by commercial kits. *P < 0.05.

As shown in Figure 3a, the suppression of HOTAIRM1 antagonized the increase of HOTAIRM1 in SK-N-SH and SK-N-AS cells caused by MPP^+^. After MPP^+^ treatment, the cell proliferation of SK-N-SH and SK-N-AS cells was decreased, while inhibition of HOTAIRM1 neutralized these effects (Figure 3b and c). Furthermore, the silencing of HOTAIRM1 functioned as the protective effect in SK-N-SH and SK-N-AS cells treated with MPP^+^ by repressing apoptosis (Figure 3d). The data of western blot assay suggested that the increased C-caspase3/t-caspase3 and Bax, as well as decreased Bcl-2 in SK-N-SH and SK-N-AS cells by treatment with the MPP^+^, were restored by silencing of HOTAIRM1 (Figure 3e and f). MPP^+^ also induced inflammation and oxidative damage by increasing TNF-α, IL-1β, ROS, LDH, and MDA levels while decreasing the SOD content, which was inverted by inhibition of HOTAIRM1 in SK-N-SH and SK-N-AS cells (Figure 3g–l). In summary, the silencing of HOTAIRM1 exerted protective effects on neuroblastoma cells against MPP^+^.

### 3.4 Overexpression of HOTAIRM1 abolished the effects of propofol on neuroblastoma cells treated with MPP^+^

**Figure 4:**
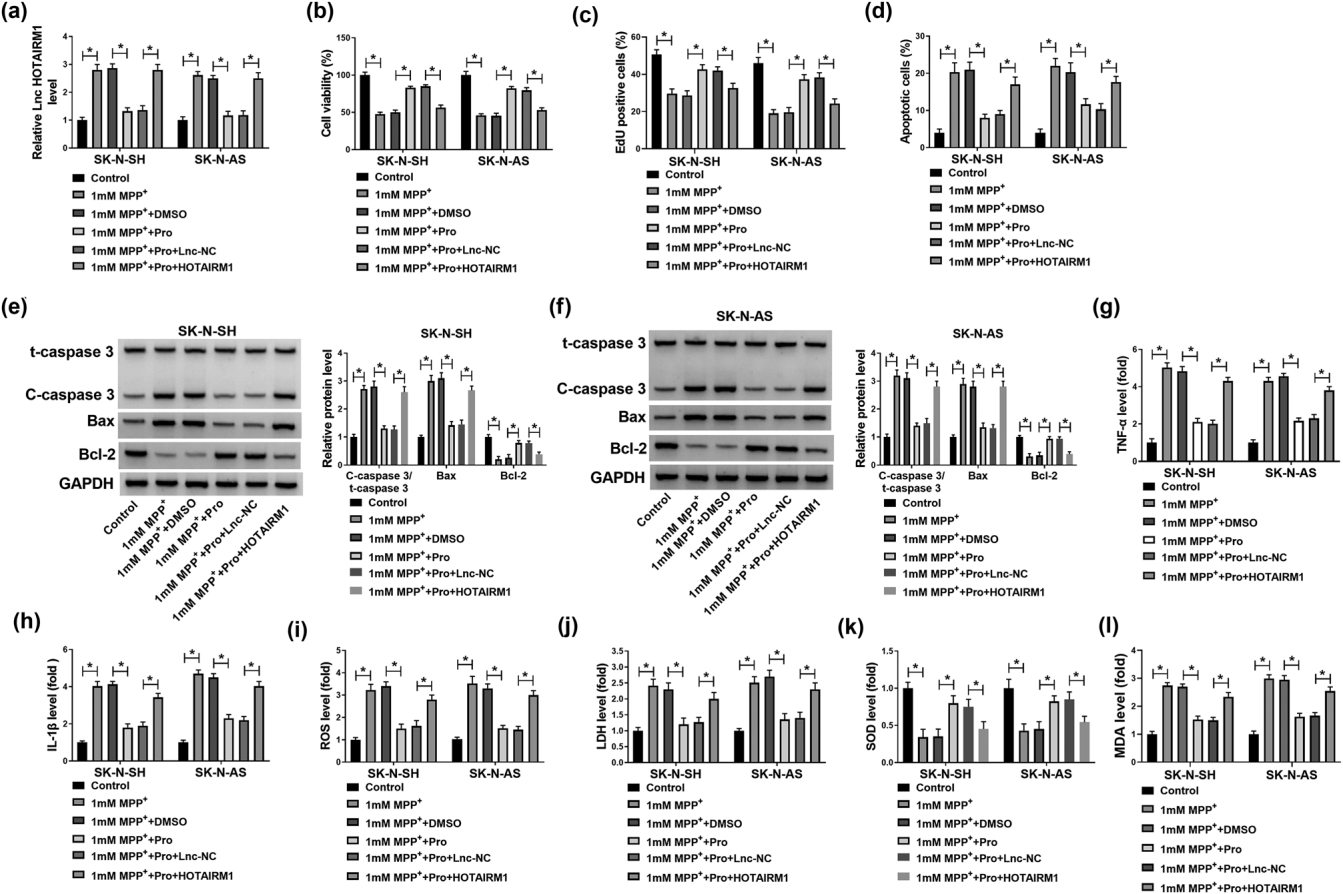
Effects of HOTAIRM1 overexpression on proliferation, apoptosis, inflammation, and oxidative stress in neuroblastoma cells treated with MPP^+^ and propofol. (a–j) SK-N-SH and SK-N-AS cells were treated with 1 mM MPP^+^, 1 mM MPP^+^ + DMSO, 1 mM MPP^+^ + Pro, 1 mM MPP^+^ + Pro + Lnc-NC, and 1 mM MPP^+^ + Pro + HOTAIRM1, with untreated cells as the Control group. (a) The expression level of HOTAIRM1 was analyzed in SK-N-SH and SK-N-AS cells by RT-qPCR assay. (b and c) MTT and EdU assays were conducted to evaluate cell proliferation. (d) Flow cytometry assay was performed to monitor apoptotic cells. (e and f) The expression levels of C-caspase3/t-caspase3, Bax, and Bcl-2 were assessed by western blot assay. (g–l) The levels of TNF-α, IL-1β, ROS, LDH, SOD, and MDA were detected by commercial kits. *P < 0.05.

The functional roles of HOTAIRM1 overexpression were investigated in SK-N-SH and SK-N-AS cells after treatment with 1 mM MPP^+^ and 50 µM of propofol. The downregulation of HOTAIRM1 in SK-N-SH and SK-N-AS cells treated with MPP^+^ and propofol was eliminated by transfection with HOTAIRM1 (Figure 4a). In addition, increased cell proliferation following propofol treatment in MPP^+^-induced cells was abrogated by HOTAIRM1 overexpression in SK-N-SH and SK-N-AS cells (Figure 4b and c). Flow cytometry data showed that propofol inhibited cell apoptosis in SK-N-SH and SK-N-AS cells treated with MPP^+^; however, the apoptosis was enhanced by transfecting with HOTAIRM1 in SK-N-SH and SK-N-AS cells (Figure 4d). The increased expression of Bcl-2 and decreased expression of C-caspase3/t-caspase3 and Bax induced by treatment with propofol were overturned by overexpression of HOTAIRM1 in MPP^+^-exposed SK-N-SH and SK-N-AS cells (Figure 4e and f). In addition, propofol could weaken MPP^+^-induced inflammation and oxidative damage in SK-N-SH and SK-N-AS cells by the regulation of TNF-α, IL-1β, ROS, LDH, SOD, and MDA, while overexpression of HOTAIRM1 neutralized the effects of propofol (Figure 4g–l). Collectively, the upregulation of HOTAIRM1 abolished propofol-induced protective effects on SK-N-SH and SK-N-AS cells exposed to MPP^+^.

### 3.5 HOTAIRM1 regulated miR-519a-3p expression in neuroblastoma cells

**Figure 5:**
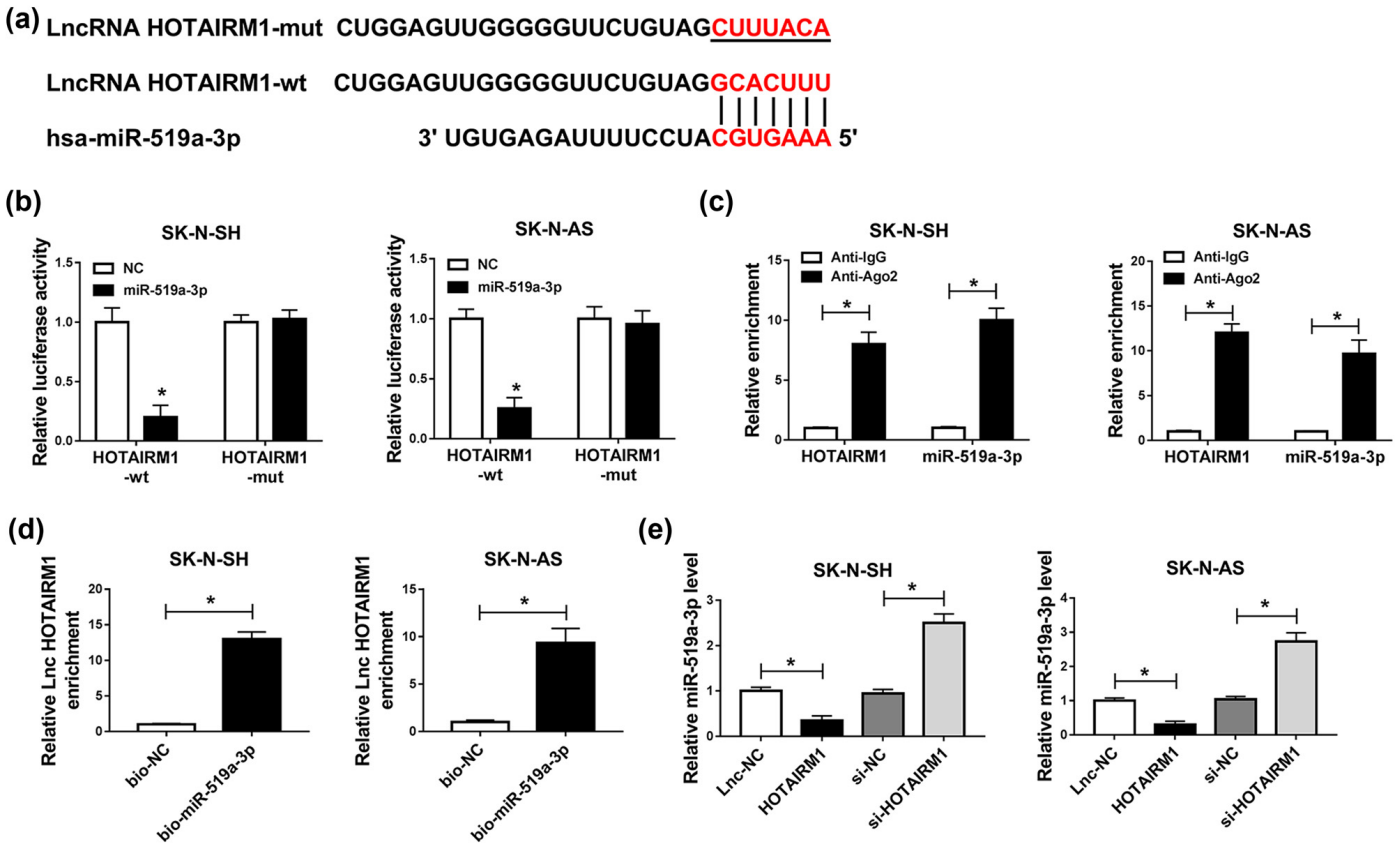
miR-519a-3p was a direct target of HOTAIRM1. (a) The possible binding region between HOTAIRM1 and miR-519a-3p is shown. (b–d) The interaction relationship between HOTAIRM1 and miR-519a-3p was confirmed by dual-luciferase reporter, RIP, and RNA pull-down assays. (e) The expression level of miR-519a-3p was examined by RT-qPCR assay in SK-N-SH and SK-N-AS cells transfected with Lnc-NC, HOTAIRM1, si-NC, or si-HOTAIRM1. *P < 0.05.

Previous results had confirmed that HOTAIRM1 and miR-519a-3p exerted important functions in SK-N-SH and SK-N-AS cells. Interestingly, HOTAIRM1 had the binding region in miR-519a-3p, as shown in Figure 5a. Furthermore, reduced luciferase activity by miR-519a-3p mimic was observed in the HOTAIRM1-wt group, while miR-519a-3p mimic did not affect the luciferase activity in the HOTAIRM1-mut group compared with control (Figure 5b). We also noticed that HOTAIRM1 and miR-519a-3p were enriched in an anti-Ago2 group compared with the anti-IgG group (Figure 5c). After the RNA pull-down assay, HOTAIRM1 exhibited higher enrichment in the bio-miR-519a-3p group than that in the bio-NC group (Figure 5d). Importantly, overexpression of HOTAIRM1 inhibited miR-519a-3p expression, while the silencing of HOTAIRM1 increased miR-519a-3p expression in SK-N-SH and SK-N-AS cells (Figure 5e). Conclusively, miR-519a-3p was negatively regulated by HOTAIRM1 in SK-N-SH and SK-N-AS cells.

### 3.6 HOTAIRM1/miR-519a-3p axis regulated proliferation, apoptosis, inflammation, and oxidative stress in neuroblastoma cells treated with MPP^+^

**Figure 6:**
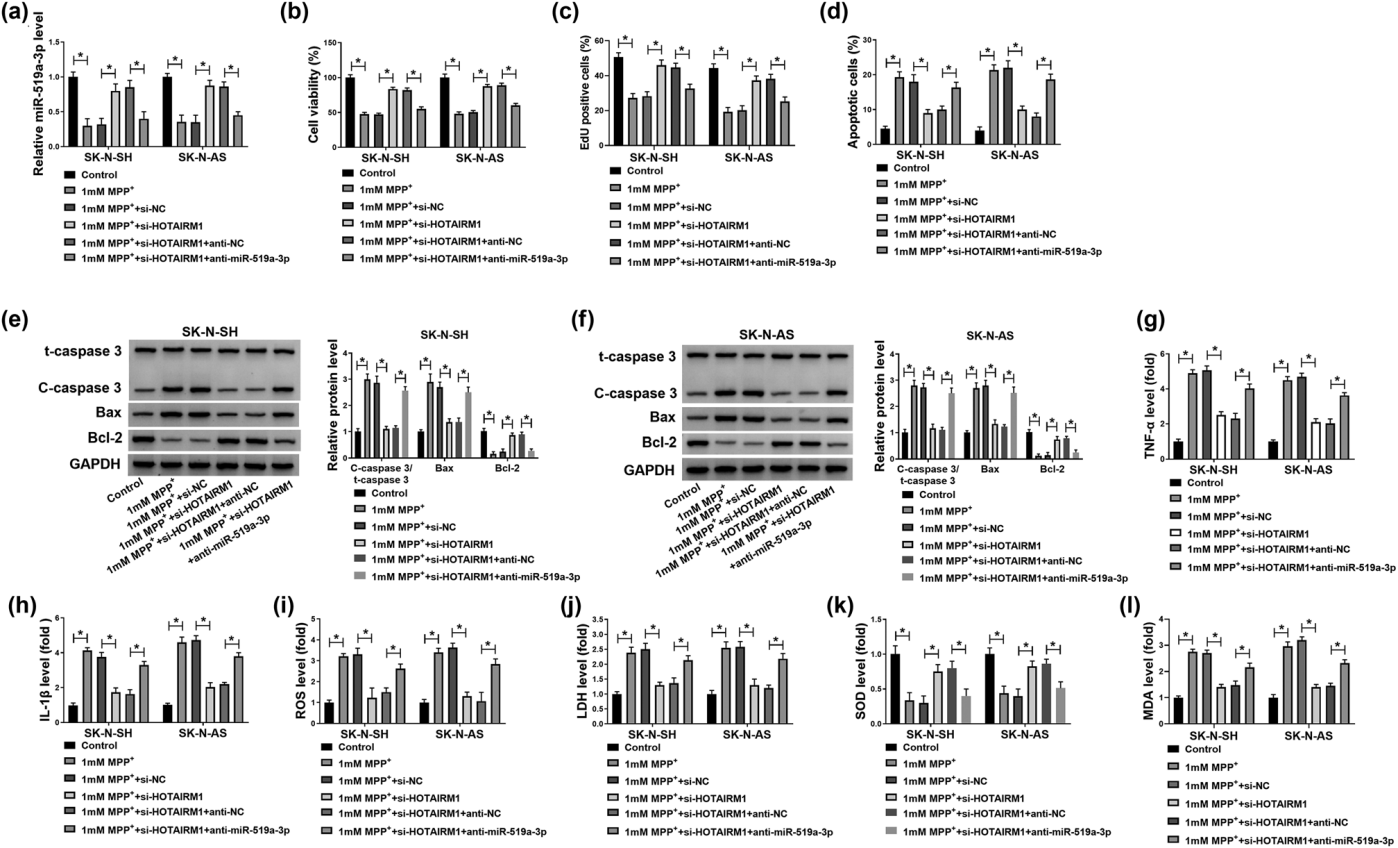
Knockdown of HOTAIRM1-induced effects on neuroblastoma cells treated with MPP^+^ was overturned by miR-519a-3p inhibition. (a–j) SK-N-SH and SK-N-AS cells were treated with 1 mM MPP^+^, 1 mM MPP^+^ + si-NC, 1 mM MPP^+^ + si-HOTAIRM1, 1 mM MPP^+^ + si-HOTAIRM1 + anti-NC, and 1 mM MPP^+^ + si-HOTAIRM1 + anti-miR-519a-3p, with untreated cells as the Control group. (a) The RT-qPCR assay was used to show the expression of miR-519a-3p. (b and c) The proliferation of SK-N-SH and SK-N-AS cells was assessed by MTT and EdU assays. (d) Flow cytometry assay was carried out to examine apoptotic cells. (e and f) The western blot assay was introduced to show the expression of C-caspase3/t-caspase3, Bax, and Bcl-2 in SK-N-SH and SK-N-AS cells. (g–l) The commercial kits were used to assess the levels of TNF-α, IL-1β, ROS, LDH, SOD, and MDA in SK-N-SH and SK-N-AS cells. *P < 0.05.

To determine the functions of HOTAIRM1 and miR-519a-3p in neuroblastoma cells, SK-N-SH and SK-N-AS cells were transfected with si-HOTAIRM1 or anti-miR-519a-3p to knock down their expression. As shown in Figure 6a, the absence of HOTAIRM1 increased miR-519a-3p expression in SK-N-SH and SK-N-AS cells exposed to MPP^+^, which was inverted by the miR-519a-3p inhibitor. MTT and EdU results indicated that suppression of miR-519a-3p counteracted the promoting role of si-HOTAIRM1 on cell proliferation (Figure 6b and c). Moreover, inhibition of miR-519a-3p reversed the suppressive effects of HOTAIRM1 silencing on cell apoptosis in SK-N-SH and SK-N-AS cells exposed to MPP^+^ (Figure 6d). Western blot assay confirmed that the silencing of HOTAIRM1 increased the expression of Bcl-2 while it decreased the expression of C-caspase3/t-caspase3 and Bax in MPP^+^-induced cells, which were overturned by silencing of miR-519a-3p (Figure 6e and f). The ELISA analysis demonstrated that the downregulation of miR-519a-3p significantly abolished si-HOTAIRM1-induced suppressive effects on TNF-α and IL-1β expression (Figure 6g and h). In addition, MPP^+^-induced oxidative damage was weakened by silencing of HOTAIRM1, while the insufficiency of miR-519a-3p abolished the effects induced by HOTAIRM1 inhibition (Figure 6i–l). All data implied that HOTAIRM1/miR-519a-3p axis played key roles in proliferation, apoptosis, inflammation, and oxidative stress of neuroblastoma cells treated with MPP^+^.

### 3.7 Inhibition of miR-519a-3p abolished propofol-induced effects on neuroblastoma cells treated with MPP^+^

**Figure 7:**
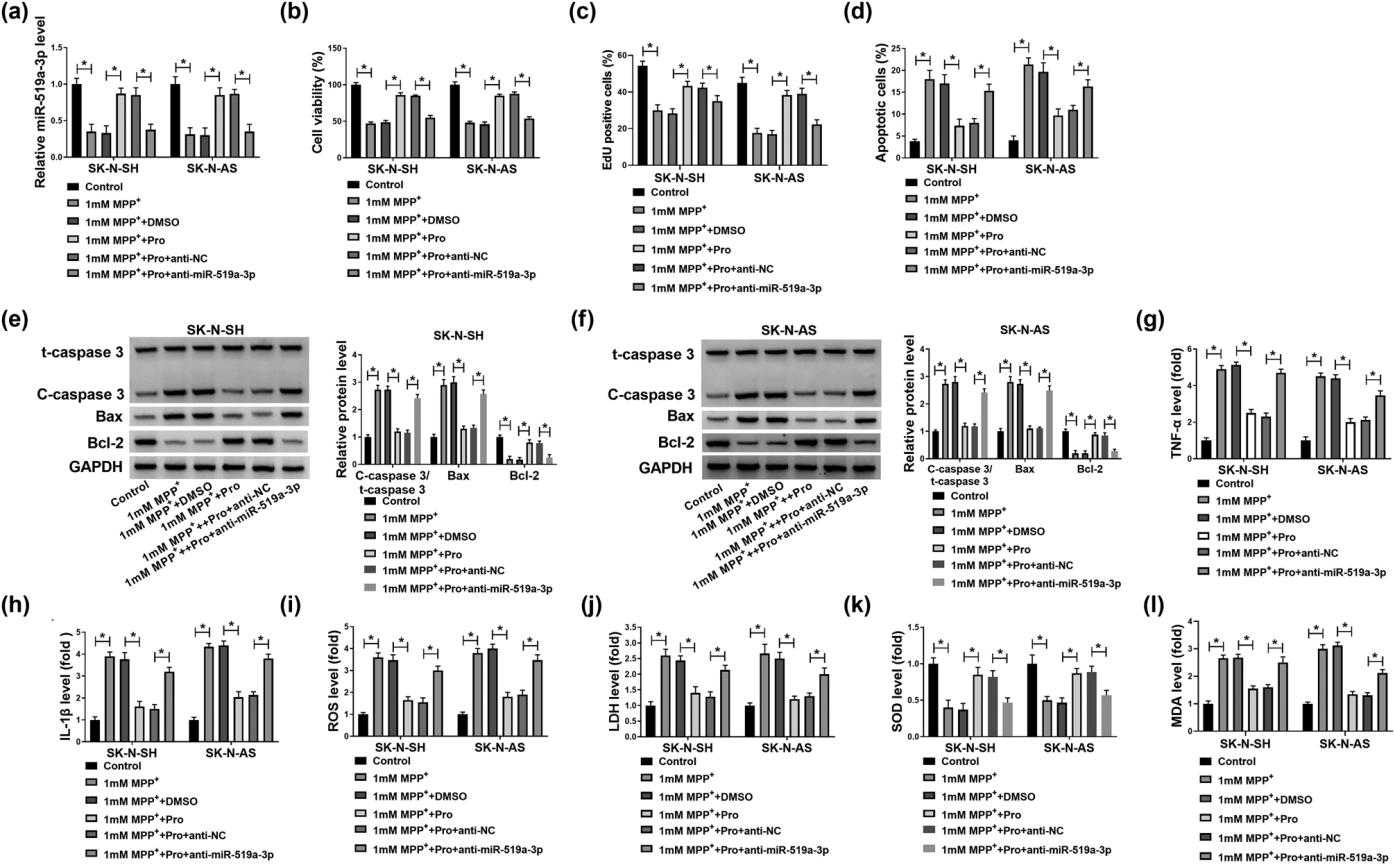
Knockdown of miR-519a-3p abolished propofol-induced effects on neuroblastoma cells treated with MPP^+^. (a–j) SK-N-SH and SK-N-AS cells were treated with 1 mM MPP^+^, 1 mM MPP^+^ + DMSO, 1 mM MPP^+^ + Pro, 1 mM MPP^+^ + Pro + anti-NC, and 1 mM MPP^+^ + Pro + anti-miR-519a-3p, with untreated cells as the Control group. (a) The expression of miR-519a-3p was assessed by RT-qPCR assay in SK-N-SH and SK-N-AS cells. (b and c) MTT and EdU assays were conducted for examining the proliferation of SK-N-SH and SK-N-AS cells. (d) Flow cytometry assay was performed in SK-N-SH and SK-N-AS after transfection. (e and f) The protein expression of C-caspase3/t-caspase3, Bax, and Bcl-2 was quantified by western blot assay. (g–l) The commercial kits were applied to show the levels of TNF-α, IL-1β, ROS, LDH, SOD, and MDA. *P < 0.05.

As displayed in Figure 7a, the upregulation of miR-519a-3p induced by propofol was abolished by miR-519a-3p inhibitor. In addition, the proliferation of SK-N-SH and SK-N-AS cells exposed to MPP^+^ was increased by propofol, which was overturned by miR-519a-3p inhibition (Figure 7b and c). The cell apoptosis was also remarkably repressed by propofol while enhanced by inhibition of miR-519a-3p in SK-N-SH and SK-N-AS cells treated with MPP^+^ (Figure 7d). The western blot assay suggested that suppression of miR-519a-3p reversed the upregulation of Bcl-2 and the downregulation of C-caspase3/t-caspase3 and Bax induced by treatment with propofol in MPP^+^-induced SK-N-SH and SK-N-AS cells (Figure 7e and f). The decrease of TNF-α, IL-1β, ROS, LDH, and MDA, as well as the increase of SOD in MPP^+^-induced SK-N-SH and SK-N-AS cells caused by propofol were inverted by suppression of miR-519a-3p (Figure 7g–l). In all, we could conclude that silencing of miR-519a-3p abolished propofol-induced effects on neuroblastoma cells treated with MPP^+^.

### 3.8 HOTAIRM1/miR-519a-3p axis regulated proliferation, apoptosis, inflammation, and oxidative stress in neuroblastoma cells

**Figure 8:**
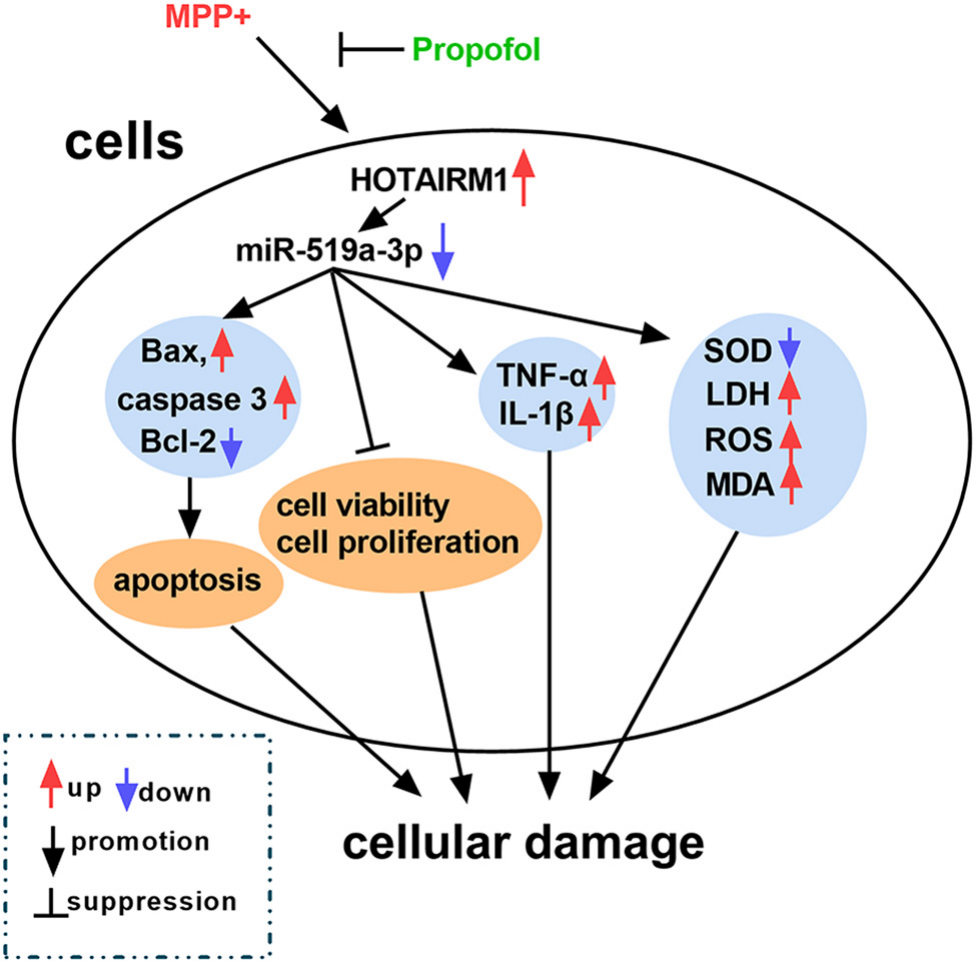
Schematic diagram of our results is shown. The regulatory networks of the HOTAIRM1/miR-519a-3p axis involved in MPP^+^ and propofol-mediated proliferation, apoptosis, inflammation, and oxidative stress are shown in neuroblastoma cells.

As shown in Figure 8a, MPP^+^ induced apoptosis, inflammation, and oxidative damage in SK-N-SH and SK-N-AS cells. Whereas propofol could counteract the effects of MPP^+^ on cells, which were involved in the HOTAIRM1/miR-519a-3p axis. Therefore, propofol may suppress apoptosis, inflammation, and oxidative damage in neuroblastoma cells treated with MPP^+^ by regulating the HOTAIRM1/miR-519a-3p axis.

## 4 Discussion

In this study, we found that propofol protected against MPP^+^-induced proliferation arrest, apoptosis, and oxidative damage in neuroblastoma cells. Mechanistically, HOTAIRM1/miR-519a-3p formed a feedback loop in cells, and silencing of HOTAIRM1 suppressed MPP^+^-induced injury by upregulating miR-519a-3p. Moreover, the HOTAIRM1/miR-519a-3p axis could be affected by propofol, and the rescue experiments indicated that propofol showed neuroprotective properties in neuroblastoma cells by regulating the HOTAIRM1/miR-519a-3p axis.

Propofol, an intravenous sedative-hypnotic agent, has antioxidant properties dependent on a phenolic structure similar to α-tocopherol, which implies the possible neuroprotective properties of propofol in PD [22,23]. Consistent with this hypothesis, the neuroprotective properties of propofol were confirmed by Kotani et al. [24]. As well known, active oxidative stress was found in PD, along with an increase of pro-oxidants in many brain areas [25]. Not surprisingly, Wang et al. revealed that propofol treatment attenuated MPP^+^-induced oxidative stress via inhibiting programmed cell death [26]. The analogous results that propofol presented a protective function for MPP^+^-induced oxidative stress in neuroblastoma cells were confirmed in this study. Furthermore, anti-inflammatory properties of propofol were also mentioned in other diseases [27,28]. Currently, ELISA analysis indicated that propofol treatment suppressed inflammation in neuroblastoma cells exposed to MPP^+^ by decreasing the levels of TNF-α and IL-1β. Besides, our study also showed that propofol treatment was beneficial to the survival of neuroblastoma cells under MMP^+^ presence. All these data implied the therapeutic potential of propofol in the PD process.

HOTAIRM1 has been shown to be a salient cancer-related lncRNA abnormally expressed in various cancers and can regulate cancer progression by affecting cell proliferation, apoptosis, invasion, and migration [29,30,31]. Besides, the regulatory effects of the HOTAIRM1-HOXA1 axis on hematopoiesis, leukemogenesis, and immunosuppressive functions were also reported [32,33]. In addition, HOTAIRM1 plays important role in regulating neuronal differentiation [34]. It was a tumor suppressor in glioblastoma multiform, and downregulation of HOTAIRM1 usually inhibited tumor growth and invasion by regulating HOXA1 expression [35]. Recently, a previous study showed that HOTAIR long noncoding RNA promoted propofol-induced neuronal pyroptosis by targeting miR-455-3p/NLRP1 axis [36]. Importantly, Fan et al. reported that dysregulated HOTAIRM1 was contributed to apoptosis of neuroblastoma cells in PD [14]. Therefore, we speculated that HOTAIRM1 might be involved in the PD process by propofol. In this study, HOTAIRM1 was upregulated in neuroblastoma cells in response to MPP^+^ treatment, while propofol abolished this effect. Functionally, knockdown of HOTAIRM1 abolished MPP^+^-induced injury neuroblastoma cells, and propofol protected against MMP^+^-induced neuroblastoma cell injury by repressing HOTAIRM1. In this study, miR-519a-3p was confirmed as a target miRNA for HOTAIRM1. miR-519a-3p was demonstrated to suppress the growth, invasion, and migration via PARP1 in ovarian cancer [37], and miR-519a-3p served as a target of LINC01419 to repress the proliferation and metastasis of osteosarcoma cells via PDRG1 [38]. Moreover, miR-519a-3p was discovered to hinder MPP^+^-evoked apoptosis and inflammation in neuroblastoma cells via the NEAT1/miR-519a-3p/SP1 axis [39]. Thereafter, our work confirmed that miR-519a-3p inhibition weakened the effects of propofol treatment or HOTAIRM1 silencing in neuroblastoma cells. Thus, a propofol/HOTAIRM1/miR-519a-3p regulatory network was identified in regulating neuroblastoma cell injury.

It was well accepted that miRNA could regulate the development of cancer biology by regulation of target mRNAs [40]. However, the involvement downstream targets of the HOTAIRM1/miR-519a-3p axis are unknown. Previous studies also showed the downstream target mRNAs of miR-519a-3p in multiple diseases, such as p53 and DNA-damage regulated 1 and TRAIL-R2 (TNFRSF10B) [18,38]. In addition, a number of signal pathways were correlated with the development of PD [41]. Nevertheless, the lack of target mRNA assay of miR-519a-3p is a certain limitation in the study, which will be explored in future experiments. Additionally, a previous study showed that the neuroprotective effect of propofol is attributed to its antioxidant properties, the inhibition of synaptic transmission mediated by the potentiation of gamma-aminobutyric acid type A (GABA(A)), and the suppression of glutamate release [42,43]. Propofol, as an intravenous sedative-hypnotic agent, is already in use clinically in neurosurgical anesthesia [43]. Thus, we speculate that the combination of propofol with RNA-targeted therapy may be beneficial for PD prevention in the clinic, which also needs further investigation in further research.

Currently, we explored the contribution of propofol in neuroblastoma cells exposed to MPP^+^, which was attributed to its association with HOTAIRM1 and miR-519a-3p, expanding its clinical application due to the beneficial properties of propofol.

In summary, propofol exerted its function as antioxidants in neuroblastoma cells treated with MPP^+^. Importantly, the regulatory roles of propofol on proliferation, apoptosis, and inflammation in neuroblastoma cells treated with MPP^+^ were involved in the HOTAIRM1/miR-519a-3p axis.
